# Measuring the extent and nature of use of Social Networking Sites in Medical Education (SNSME) by university students: Results of a multi-center study

**DOI:** 10.1080/10872981.2018.1505400

**Published:** 2018-08-07

**Authors:** Salman Yousuf Guraya, Hamdi Almaramhy, Mona Faisal Al-Qahtani, Shaista Salman Guraya, Manal Bouhaimed, B. Bilal

**Affiliations:** a Surgery Clinical Sciences Department, College of Medicine, University of Sharjah, Sharjah, UAE; b Dean College of Medicine, Taibah University Almadinah Almunawwarah, Saudi Arabia; c Department of Public Health, College of Public Health, Imam Abdulrahman Bin Faisal University, Saudi Arabia; d Medical Education Unit, College of Medicine University of Sharjah, United Arab Emirates; e Department of Community Medicine and Behavioural Sciences, Department of Surgery, Faculty of Medicine, Kuwait University, The State of Kuwait; f School of Accountancy, Hubei University of Economics, Wuhan, China

**Keywords:** Social networking sites, Facebook, Twitter, Flickr, education

## Abstract

There is a sharp rise in the use of social networking sites (SNSs) by university students for various purposes. However, little is known about the use of SNSs for educational purposes. This study aims to determine educational use of SNSs by the medical students of two Saudi and a Kuwaiti medical school. A cross-sectional study was conducted by administering a 20-statement questionnaire to the undergraduate medical and allied health sciences students of two Saudi universities: Taibah University (TU) and Imam Abdulrahman AlFaisal University (IAFU), and one Kuwaiti university, Kuwait University (KU). The data were collected and analyzed by SPSS 20. Of a total of 1312 respondents, 1181 (90%) students used SNSs and 131 (10%) did not use SNSs for any reason. Further, only 442/1181 (37%, *p* < 0.00) students used SNSs for education and found these sites to be useful. As many as 357 (27%) students used SNSs for sharing education-related information once a day, 306 (23%) 3–5 times a day, and 331 (25%) once a week. A maximum of 678 (52%) used Facebook for educational purpose and most of the students, 469 (38%), used SNSs for sharing lectures. There were significant variations in responses among genders and year of schooling. The use of online social media in medical education is a rapidly evolving arena of scholarship. Low use of SNSs for sharing information and significant variations in perceptions of medical students about social media should draw attention of policy-makers for promoting awareness and educational reforms.

## Introduction

Developments of cutting-edge technologies and the advent of computer and Internet have exponentially increased use of Internet-based medical transformation [1]. The growing application of Internet-based learning in health professions education has been initially perceived as an effective platform for timely, convenient, and collaborative education [2]. One of the groundbreaking innovations in the Internet-based platforms with multidimensional purposes is development of social networking sites (SNSs). ‘The SNSs are web-based platforms on which members can create personal profiles, articulate friendship connections, and socially interact with the friend connections by uploading, liking, and commenting on content such as photos, messages, and videos shared on newsfeeds’ [3]. A recent study by Duggan has shown that, among the American adults, a great majority use smart phones by adopting the Web 2.0-based applications of SNSs and approximately 72% use Facebook, 31% Pinterest, 28% Instagram, 25% LinkedIn, and 23% Twitter [4]. An indicator of popularity of such SNSs is reflected by the average time spent on Facebook alone that has increased from 40 min in 2014 to 50 min in 2016 [5]. A report conducted on 455 students, residents, and physicians has shown that 94% medical students, 79.4% residents, and 42% practicing physicians used SNSs [6]. This reflects an ever-growing use of SNSs among young medical students.

The purposes of usage of SNS-based platforms are far ranging; information sharing, leisure and fun, and for teaching and learning. In terms of the educational use of SNSs, students use these platforms for putting together presentations, timetabling and using calendar of activities, developing collaborative group work, group discussions, facilitating and evaluating learning, faculty support, and for updating regional and global knowledge. A systematic review and meta-analysis by Guraya [7] has reported that 20% of the university medical students used SNSs in sharing information for educational purposes. However, the study could not find any study that envisaged to determine the impact of SNSs on the students’ academic performance. Several forms of online social media are used by medical students during their quest for knowledge gain; *blogging*, a teaching tool for healthcare clients that can facilitate knowledge sharing among students, physicians, patients, and administrators; *microblogging*, by Twitter account for instance, is used for communicating with a broader audience, coaching medical students, sending links, and experts opinions; *wikis*, used for collaborative writing, interprofessional education among medical students, physicians, nurses, researchers, and academia; *audio/video* sharing by podcasts; for presentations and for general healthcare issues; *social bookmarking and tag clouds*, for facilitating information about areas of common research interest among communities [8]. At the same time, the innovative technologies in digital world have transformed the landscape of educational trends using smartphones and tablets for QR codes, virtual learning, and gamification in courses [9].

There is some evidence that the medical students use SNSs for education, but there are little data that can explicitly determine the degree and nature of use of SNSs in medical education. This study aimed to determine the extent and nature of use of SNSs by the medical students from a Kuwaiti and two Saudi medical schools.

## Materials and methods

### Study settings

This study was conducted in medical and allied health sciences of two Saudi Universities. Taibah University (TU) and Imam Abdulrahman AlFaisal University (IAFU), and one Kuwaiti university, Kuwait University (KU). The medical college of TU follows a 5-year MBBS program with a competency-based problem-based learning-integrated spiral curriculum. The existing curriculum contains fundamental strands of early clinical experience, personal professional portfolios, student-directed learning, portfolios, blended learning, and clinical reasoning. All leaners are provided with a rich E-learning platform that strengthens their learning ability and enhance their knowledge and competency framework at their own pace. At IAFU, the MBBS curriculum applies interdisciplinary approach with horizontal and vertical integration of biomedical and clinical skills sciences. The learning strategies include problem-based, case-based, community-based, and patient-centered learning activities. The students are engaged in active learning with early clinical exposure where elective modules are offered to the students [10]. KU employs a system-based case-triggered integrated strategy in three phases; phase I contains first two semesters of the pre-professional program, phase II covers semesters 3–8 of the medical curriculum. The core principles applied are system-based, student-centered approaches with emphasis on self-directed learning and student motivation. The students who successfully complete phase II program are awarded the B.Med. Sc. Degree that is prerequisite for admission to the clinical program (phase II) that trains students in clinic settings.

### Study design

During October to November 2016, a cross-sectional study was conducted on the first year through fifth-year undergraduate medical and allied health sciences students of TU, IAFU, and KU. Ethical approval was obtained by the institutional review boards of respective universities. A 20-statement validated English-language questionnaire, Social Networking Sites in Medical Education (SNSME), was administered to all participants. The first 6 statements of the questionnaire enquired about frequency of the usage of SNSs by giving five options of never, once a month, once a week, once a day, and 3–5 times per day. The next 14 statements of the questionnaire were used to capture the opinions of participants about the usage of SNSs for educational purposes on a 5-point Likert scale (e.g., strongly agree, agree, neutral, disagree, and strongly disagree). SurveyMonkey_®_ was used for the administration of instrument and collection of data in TU and KU and a paper-based survey was conducted in IAFU.

### Data collection and analysis

The data were entered and analyzed through Statistical Package for Social Sciences v.20 (SPSS). The descriptive analysis was done by frequency distribution and graphical pictorial presentation was shown in clustered bar charts. As all statements were arranged in ordinal scale, inferential statistics were performed by non-parametric tests. The non-parametric *χ*^2^ test was applied for the analysis of variables (statements), arranged in categorical format. The *χ*^2^ test would explore the differences between observed frequencies and expected frequencies within each statement. As a pre-requisite to using other non-parametric tests (Mann–Whitney *U* and Kruskal–Wallis tests), the normality of data was verified by one-sample Kolmogorov–Smirnov test. If a variable has significant *p* value <0.05, this would reject the null hypothesis that ‘data is normally distributed’. Henceforth, the non-parametric tests would be deemed appropriate for comparison of responses based on gender, year of schooling and age groups of the respondents. The Mann–Whitney *U* test was used to compare the differences between genders, and the Kruskal–Wallis test was applied to compare the differences between more than two independent groups such as year of schooling and age groups of students. A *p* value ≤0.05 was considered significant.

## Results

Of 1986 invitees, we received 1312/1986 complete responses (*N* = 1312, response rate of 66%): 746 (57%) female and 566 (43%) male students. The data showed that there were 70 (5%) students from first year, 255 (19%) students from second year, 411 (31%) students from third year, 166 (13%) students from fourth year, and 408 (31%) fifth-year students. The 510 (39%) students were in 18–24 years, majority of respondents 793 (60%) were in age group 25–34 years, and 9 (0.7%) students were older than 34 years. Overall, 1181 (90%) students used and 131 (10%) did not use SNSs for any reason. Of those who used SNSs, only 442/1181 (37%) students pointed out that they were using SNSs for educational purposes and had found these sites to be useful. As many as 206/796 (26%) IAFU students, 156/352 (44%) TU students, and 80/164 (49%) KU students strongly agreed that SNSs were useful for educational purposes.

**Figure 1.**
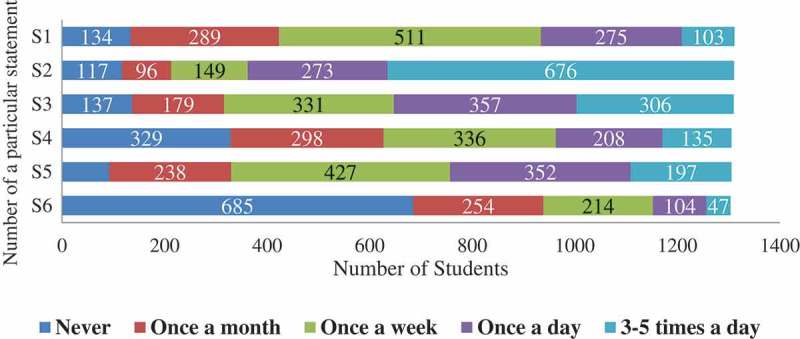
The observed frequencies of responses to statements about the students’ extent of use of Social Networking Sites for Medical Education (SNSME) (*N* = 1312).

Figure 1 shows the clustered bar chart of the observed frequencies in responses to statements regarding the students’ extent of usage of SNSs for educational purpose through a categorical variable (e.g., never used, once a month, once a week, once a day, and 3–5 times a day). Regarding the statement ‘how often do you use e-mail for sharing information for educational purpose?’ we observed that the majority 511 (39%) used e-mail once a week for sharing educational material. For the statement ‘how often do you use social networking sites to keep in touch with peers and tutors?’ 676 (52%) students used SNSs to remain in touch with their peers and tutors, for ‘how often do you use social networking sites (e.g., Facebook, Youtube, Twitter, Linkedin, and Flickr) to share education-related information?’ 357 (27%) students used SNSs for sharing education-related information once a day, 306 (23%) students 3–5 times a day, and 331 (25%) students share once a week, and for ‘how often do you contribute to blogs or Wikis to share information, or disseminate knowledge?’ most of the students 685 (52%) did not contribute in blogs writing. Overall, most of the students used SNSs once a week for educational purposes as shown in Figure 1 (S1, S4, and S5).

**Figure 2.**
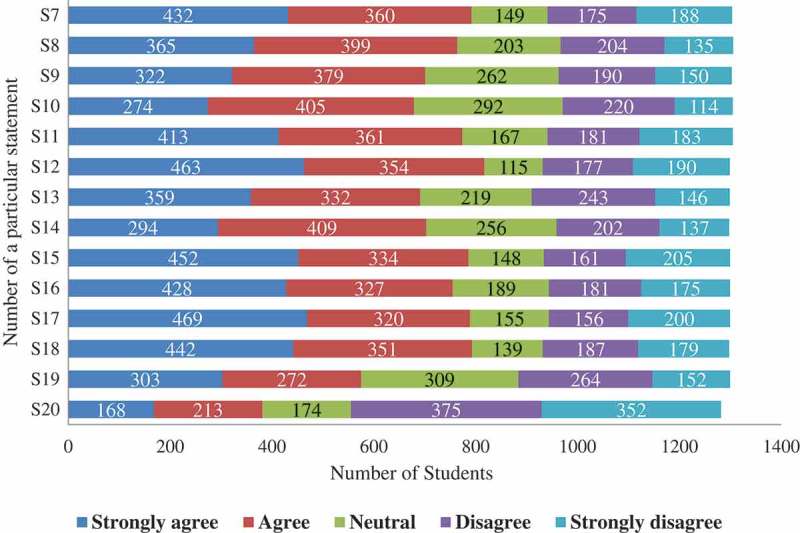
The observed frequencies of responses to statements about the students’ perceptions of use of Social Networking Sites for Medical Education (SNSME) (*N* = 1312).

Figure 2 displays the bar chart of the observed frequencies of responses to statements about the students’ usage of the SNSs for educational purpose using a 5-point Likert scale (e.g., strongly agree, agree, neutral, disagree, and strongly disagree). The highest response was recorded for the 17th statement ‘I have found social networking sites useful for sharing notes and lectures’, as a majority of 469 (36%) students strongly agreed that SNSs was an important platform for sharing educational material. On the other hand, for the 20th statement ‘I believe that social networking sites are inappropriate for sharing classroom materials, information, and discussing education related topics’, 375 (29%) respondents disagreed and 352 (27%) disagreed, respectively.

The results of the *χ*^2^ test (*χ*^2^(4, *N* = 1312) = 62~968, *p* < 0.0001) showed that all statements were significant reflecting that the observed frequencies of student’s responses are statistically significant from expected frequencies within each category (Table 1). The results of Kolmogorov–Smirnov test confirm that data are not normally distributed as each statement has significant *p* < 0.0001 (untabulated). Thus, the non-parametric tests, Mann–Whitney *U* test and Kruskal–Wallis test, were deemed appropriate for the comparison of responses to statements on the basis of gender, year of schooling, and age groups of students. The Mann–Whitney *U* test was used to compare the responses of male and female students towards extent of usage and perception of students about SNSs for educational purposes as shown in Table 2. The results of Mann–Whitney *U* test showed significant variations in responses to all statements among male and female students that are significant at *p* < 0.0001 except for the statement 4 with *p* = 0.09. The responses by female students scored significantly higher mean ranks to first 5 statements about the extent and use of SNSs for education and to statement 20: for statement 1, a highest mean rank of 733 was noted for female students (Median = 4) as compared to mean rank of 555 for male students (Median = 3), *U* = 153,969, *p* < 0.0001, *r* = 0.24. On the other hand, for statements 6–19, male students scored higher mean ranks: male students (Median = 3) for statement 15 recorded a highest mean rank of 824.47 than 520 mean rank for female students (Median = 2), *U* = 110,107, *p* < 0.0001, *r* = 0.41.

**Table 1.** Perceptions about use of social networking sites in study cohort by *χ*^2^ test using Social Networking Sites for Medical Education (SENSME) (*N* = 1312).

Statements	*χ*^2^	*p* values
1. How often do you use e-mail for sharing information for educational purpose?	398^a^	0.00*
2. How often do you use social networking sites (e.g., Facebook, YouTube, Twitter, LinkedIn, and Flickr) to keep in touch with peers and tutors?	888^b^	0.00*
3. How often do you use social networking sites (e.g., Facebook, YouTube, Twitter, LinkedIn, and Flickr) to share education-related information?	146^c^	0.00*
4. How often do you use social networking sites for sharing research, innovations in medical field?	116^d^	0.00*
5. How often do you read blogs or Wikis for education-related information?	264^d^	0.00*
6. How often do you contribute to blogs or Wikis to share information, or disseminate knowledge?	968^e^	0.00*
7. Social networking sites help me in collection of educational materials	247^e^	0.00*
8. Social networking sites are helpful in collaborative and peer-to-peer learning	200^d^	0.00*
9. Social networking sites are useful in developing reading and writing web skills	134^f^	0.00*
10. Social networking sites provide opportunity of virtual meetings with other students and tutors	173^g^	0.00*
11. Social networking sites help me to communicate with peers about class projects	208^g^	0.00*
12. Social networking sites help me to access educational resources	319^h^	0.00*
13. Social networking sites help me to retrieve educational references for research	115^h^	0.00*
14. Social networking sites facilitate my professional development of technological skills	161^i^	0.00*
15. Social networking sites are useful in communicating with classmates about course-related topics	260^j^	0.00*
16. I have found social networking sites useful during the pre-exam period when I get an instant answer/explanation from my peer, instead of going through the books	197^j^	0.00*
17. I have found social networking sites useful for sharing notes and lectures	280^j^	0.00*
18. I have found social networking sites useful for educational purposes	262^i^	0.00*
19. Students need supervision and guidance for the appropriate use of social networking sites for educational purposes	62^j^	0.00*
20. I believe that social networking sites are inappropriate for sharing classroom materials, information, and discussing education-related topics	155^k^	0.00*

Note: 0 cells (.0%) have expected frequencies of less than 5. The minimum expected cell frequency is (a = 262.4, b = 262.2, c = 262, d = 261.2, e = 260.8, f = 260.6, g = 261, h = 259.8, i = 259.6, j = 260, and k = 256.4). Here * represents *p* value of <0.01.

**Table 2.** The results of the Mann–Whitney *U* test showing comparison of the students’ perceptions about use of Social Networking Sites for Medical Education (SENSME) among gender (*N* = 1312).

Statement	Male MR	Female MR	Mann–Whitney *U*	*Z*	*p* values
1	555.53	**733.11**	153,969	−8.77	0.00*
2	579.73	**713.95**	167,666	−6.89	0.00*
3	612.38	**688.10**	186,051	−3.69	0.00*
4	633.45	**668.64**	197,797	−1.71	0.09**
5	618.57	**679.88**	189,435	−3.01	0.00*
6	**712.71**	607.18	174,605	−5.46	0.00*
7	**821.72**	524.33	113,403	−14.60	0.00*
8	**820.75**	527.16	115,069	−14.36	0.00*
9	**800.98**	539.01	124,494	−12.79	0.00*
10	**797.06**	544.38	127,875	−12.34	0.00*
11	**826.01**	522.13	111,549	−14.88	0.00*
12	**807.16**	532.76	119,235	−13.54	0.00*
13	**792.20**	543.59	127,493	−12.13	0.00*
14	**805.88**	533.05	119,451	−13.35	0.00*
15	**824.47**	520.49	110,107	−14.95	0.00*
16	**806.45**	533.95	120,122	−13.35	0.00*
17	**824.12**	520.75	110,299	−14.94	0.00*
18	**821.82**	520.38	110,466	−14.84	0.00*
19	**757.87**	570.26	147,135	−9.12	0.00*
20	556.23	**707.22**	154,416	−7.45	0.00*

Note: MR: mean rank. **Note:** Grouping variable is age group. * and ** represent *p* < 0.01 and *p* < 0.05, respectively. MR: mean rank.

Bold values reflect statistically significant differences between responses by male and female participants.

Table 3 compares the responses of participants to all statements, as measured by mean ranks, across years of schooling. The results of Kruskal–Wallis test showed that the students’ responses were significantly different for all statements *p* < 0.05. The responses of fourth-year students significantly dominated those from other years as evident by their higher mean ranks, e.g., for statement 4, a significantly (*H*(4) = 33.41, *p* < 0.0001) highest mean rank of 790 by fourth year students than 651 for third year, 633 for second year, 626 for fifth year, and 534 for first year. Table 4 compares all statements on the basis of mean ranks across different age group of students and the results of Kruskal–Wallis test showed that the students’ responses were significantly different from each other for all statements *p* < 0.05. The students with age group 25–34 years scored significantly (*H*(2) = 30.03, *p* < 0.0001) highest mean rank of 698 for statement 4 than 679 for age group above 34 years and 584 for age group 18–24 years.

**Table 3.** The results of the Kruskal–Wallis test showing comparison of the students’ perceptions about use of Social Networking Sites for Medical Education (SNSME) across years of schooling (*N* = 1312).

Statement	Year 1	Year 2	Year 3	Year 4	Year 5	*χ*^2^	*p* values
1	671	**699**	579	686	690	26.98	0.00*
2	653	666	601	**745**	666	21.71	0.00*
3	551	**702**	604	686	680	20.64	0.00*
4	447	638	643	657	**705**	30.80	0.00*
5	602	613	648	638	**697**	10.70	0.03**
6	499	642	640	649	**696**	21.11	0.00*
7	511	635	661	**774**	626	32.21	0.00*
8	486	630	664	**764**	638	31.95	0.00*
9	534	633	651	**790**	626	33.41	0.00*
10	479	651	677	**733**	624	28.07	0.00*
11	503	640	666	**724**	642	19.45	0.00*
12	509	647	661	**757**	619	28.71	0.00*
13	518	668	660	**774**	597	37.57	0.00*
14	555	653	660	**758**	605	25.80	0.00*
15	466	647	668	**760**	619	37.39	0.00*
16	483	651	678	**772**	599	44.11	0.00*
17	419	662	670	**760**	616	49.09	0.00*
18	474	658	667	**758**	610	37.52	0.00*
19	645	676	644	**761**	593	26.72	0.00*
20	**828**	660	640	602	611	24.23	0.00*

Note: MR: mean rank. Here grouping variable is age group. * and ** represent *p* values of <0.01 and <0.05, respectively.

Bold values reflect statistically significant differences between responses by male and female participants.

**Table 4.** The results of the Kruskal–Wallis test showing comparison of the students’ perceptions about use of Social Networking Sites for Medical Education (SNSME) across age groups (*N* = 1312).

Statement	18–24 years	25–34 years	Above 34 years	*χ*^2^	*p* values
1	**717**	620	454	25.21	0.00*
2	668	648	**677**	1.08	0.58
3	655	655	**737**	0.45	0.80
4	584	**698**	679	30.03	0.00*
5	617	676	**766**	8.77	0.01**
6	583	697	**708**	34.07	0.00*
7	638	**663**	588	1.73	0.42
8	609	**683**	583	12.93	0.00*
9	621	**673**	588	6.49	0.04**
10	635	**665**	594	2.39	0.30
11	612	**681**	481	13.07	0.00*
12	631	**664**	548	3.36	0.19
13	648	**652**	566	0.52	0.77
14	639	**657**	615	0.77	0.68
15	624	**669**	516	5.96	0.05***
16	626	**669**	482	6.18	0.05***
17	611	**680**	375	16.47	0.00*
18	620	**670**	535	6.85	0.03**
19	**690**	625	583	9.99	0.01**
20	692	605	**884**	22.32	0.00*

Note: MR: mean rank. Here grouping variable is age group. *, **, and *** represent *p* values of <0.01, <0.05, and <0.10, respectively. Bold values reflect statistically significant differences between responses by male and female participants.

## Discussion

This study has three main findings. First, majority of the medical students at two major universities in KSA and Kuwait used SNSs, however, only a minority (37%) used SNSs for education. Second, male students used SNSs more than female students to communicate with peers. Third, there were significant variations in responses among year of schooling and the trend showed that senior students used SNSs more than junior students.

Our study has indicated that although a great majority of students used SNSs, this usage was not effectively channelized towards effective learning process and the students had varying perceptions about its usage. In our study, majority of students used SNSs once a week and few students used SNSs once a day for education. In a study by DiVall and Kirwin [11], the authors studied the use and perceptions of a comprehensive disease management course students about Facebook in facilitating online discussion of course content. The researchers have reported that the students were more engaged and intrigued to use Facebook than Blackboard for posting their educational material. In addition, the students were interested to use Facebook in future upcoming courses as well. Incorporation of such educational content into medical curricula would certainly promote active learning by the students at their own pace. Another study surveyed perceptions of the students about anatomy education using social media and has purported that the majority of respondents preferred Facebook as an effective learning tool [12].

In our study, the data about use of SNSs for educational purposes were collected without any intervention. This could be the reason for low usage of online social media by the studied cohort. This finding necessitates increasing awareness and interventions in existing curricula by embedding SNSs into instructional strategies. The value of embedding SNS-based instructional strands into medical curricula has been reaffirmed by a systematic review by Cheston et al. who have argued that interventions using SNSs foster opportunities of engaging learners, professional enhancement and effective feedback [13]. Nevertheless, the authors have cautioned the use of social media due to privacy and security issues and technical troubleshooting.

A plethora of research has convincingly shown that use of social media supplements delivery and understanding of medical education [14], lifelong learning [15], and medical professionalism [16]. The use of social media in medical education for active learning demands incorporation of scientifically designed program that should address ethics [17], facilitate group learning [18] especially at the workplace-based education [19] in an interprofessional environment [20,21]. One of the limitations of our study is the lack of social media-based educational content in the curricula. This might be due to challenges and difficulties in selecting a certain social media platform as each SNS has specific method and delivery of contents. For instance, restrictions in character limits by Twitter can often limit explanations that will enforce users to inconveniently divide text-based content over multiple tweets [22]. On the other hand, Instagram offers liberal character limits on captions, thus giving liberty for in-depth online conversations and dialogues. Gray et al. have shown that the medical students can conveniently use Facebook for discussing examination questions, posting diagrams and figures, listing tips for studying before examinations, and providing useful links to peers [23]. On the other hand, Facebook has been shown to be more frequently used for physician–patient interaction in sharing medical content with patients and families [24]. Thus, despite the existence of potential use of SNSs in medical education, there is no standard mechanism and platform that can tailor educational needs of the students.

Our study showed significant variations in responses between genders and across year of studying. These variations might reflect differing learning needs across years of study and genders as proposed in a study by Han et al. [25]. Gender differences among university students for using SNSs has been reported by other studies as well [26,27].

The findings of our study showed that the majority of the students used Facebook for education and sharing lectures and notes was the most popular mode of educational use of SNSs. On the other hand, in contrast to our study, Forgie et al. have proposed that thoughtful application of Twitter in higher education can be used as an effective adjunct to teaching and learning [28]. However, Facebook and Flickr have also been advocated for effective teaching and learning in medical education [29,30]. Such variations about use of SNSs platforms might be reflected by differences between the Western and Middle East educational environment that will influence students’ preferences. For any practical reason, Facebook, Twitter and other online social media can be customised to tailor individual learning needs and the crucial factor is to carefully embed the use of SNSs for teaching and learning in medical education. In the current study, most of the Kuwaiti students emphasized the need for supervision and guidance while using SNSs for education; whereas a great of the Saudi students have proposed a standard legislative framework for using SNSs.

The use of SNSs is not without problems; Internet addiction, cybercrimes and harassments, and less productivity are so of those [31]. A growing body of literature has argued that university students engage in several activities in SNSs to enrich and supplement their academic activities [32–34]. Such phenomenon distracts students from their primary goal of achieving high grades. These reports necessitate a controlled and supervised use of SNS for education that can potentially lead to high academic performance. Some of the users become socially reclusive due to unnecessary emphasis on virtual interaction and ignore the jest of real-world social connectivity [35]. The addiction of SNSs has been shown to adversely influence personal and work environment, increases distraction, and undermines positive emotions [36]. The habitual use of Facebook negatively affects behavioral relations [37]. Growing concerns have been raised about negative impact of SNSs such as legal and professional risks, extroversion and low conscientiousness, loneliness, personality traits and self-esteem issues [38,39]. Such information should alert policy-makers to advocate appropriate use of SNSs for education that can enrich learning climate without harming students’ behaviors and interpersonal relationships. An institutional code of conduct about academic integrity [40], adherence to core principles of medical professionalism [41] and implementing legal policies about use SNSs can potentially resolve some of the mentioned shortcomings [42].

## Conclusion

This research draws upon the perceptions of medical students from three Arabian institutions about the degree and nature of use of SNSs for their learning and education. Although a great majority of students use SNSs for various purposes, but use of such platforms for sharing educational material is low. Students have emphasized the need for sessions delineating appropriate use of SNSs in education and discussions related to legal and ethical issues surrounding SNSs in medical education. The findings of this study urge educators to work on curricular reforms that can embed social media for education.

## References

[1] Volkow N, Li T-K. The neuroscience of addiction. Nat Neurosci. 2005;8(11):1429–8. 16251981 10.1038/nn1105-1429

[2] Cook DA, Levinson AJ, Garside S, et al Internet-based learning in the health professions: a meta-analysis. Jama. 2008;300(10):1181–1196. 18780847 10.1001/jama.300.10.1181

[3] Ellison NB, Vitak J, Gray R, et al Cultivating social resources on social network sites: facebook relationship maintenance behaviors and their role in social capital processes. J Comput Mediat Commun. 2014;19(4):855–870.

[4] Ellison NB, Steinfield C, Lampe C The benefits of Facebook “friends:” Social capital and college students’ use of online social network sites. J Comput Mediat Commun. 2007;12(4):1143–1168.

[5] Ellison NB Social network sites: definition, history, and scholarship. J Comput Mediat Commun. 2007;13(1):210–230.

[6] Foley NM, Maher BM, Corrigan MA Social media and tomorrow’s medical students—how do they fit? J Surg Educ. 2014;71(3):385–390. 24797855 10.1016/j.jsurg.2013.10.008

[7] Guraya SY The usage of social networking sites by medical students for educational purposes: a meta-analysis and systematic review. N Am J Med Sci. 2016;8(7):268–278. 27583234 10.4103/1947-2714.187131 PMC4982355

[8] Popoiu MC, Grosseck G, Holotescu C What do we know about the use of social media in medical education? Procedia Soc Behav Sci. 2012;46:2262–2266.

[9] Sparks MA, O’Seaghdha CM, Sethi SK, et al Embracing the Internet as a means of enhancing medical education in nephrology. Am J Kidney Dis. 2011;58(4):512–518. 21840099 10.1053/j.ajkd.2011.06.009

[10] Abdulrahman KB, Harden R, Patrício M Medical education in Saudi Arabia: an exciting journey. Med Teach. 2012;34(sup1):S4–S5. 22409190 10.3109/0142159X.2012.660509

[11] DiVall MV, Kirwin JL Using Facebook to facilitate course-related discussion between students and faculty members. Am J Pharm Educ. 2012;76(2):32. 22438604 10.5688/ajpe76232 PMC3305941

[12] Jaffar AA Exploring the use of a Facebook page in anatomy education. Anat Sci Educ. 2014;7(3):199–208. 24022984 10.1002/ase.1404

[13] Cheston CC, Flickinger TE, Chisolm MS Social media use in medical education: a systematic review. Acad Med. 2013;88(6):893–901. 23619071 10.1097/ACM.0b013e31828ffc23

[14] Galiatsatos P, Porto-Carreiro F, Hayashi J, et al The use of social media to supplement resident medical education–the SMART-ME initiative. Med Educ Online. 2016;21(1):29332. 26750511 10.3402/meo.v21.29332 PMC4707390

[15] Kind T, Evans Y Social media for lifelong learning. Int Rev Psychiatry. 2015;27(2):124–132. 25906988 10.3109/09540261.2014.990421

[16] Gholami-Kordkheili F, Wild V, Strech D The impact of social media on medical professionalism: a systematic qualitative review of challenges and opportunities. J Med Internet Res. 2013;15(8):e184. 10.2196/jmir.2708 PMC3758042 23985172

[17] Guraya SY, London N, Guraya SS Ethics in medical research. J Microsc and Ultrastruct. 2014;2(3):121–126.

[18] Guraya SS, Guraya SY, Habib FA, et al Learning styles of medical students at Taibah University: trends and implications. J Res Med Sci. 2014;19(12):1155–1162. 25709657 10.4103/1735-1995.150455 PMC4333524

[19] Guraya SY Workplace-based assessment; applications and educational impact. Malays J Med Sci. 2015;22(6):5–10. PMC5295751 28223879

[20] Guraya SY, Barr H The effectiveness of interprofessional education in healthcare: a systematic review and meta-analysis. Kaohsiung J Med Sci. 2018;34(3):160–165. 29475463 10.1016/j.kjms.2017.12.009 PMC12977169

[21] Al-Qahtani MF, Guraya SY Measuring the attitudes of healthcare faculty members towards interprofessional education in KSA. J Taibah Univ Med Sci. 2016;11(6):586–593.

[22] Ranginwala S, Towbin AJ Use of social media in radiology education. J Am Coll Radiol. 2018;15(1,Part B):190–200. 29102536 10.1016/j.jacr.2017.09.010

[23] Gray K, Annabell L, Kennedy G Medical students’ use of Facebook to support learning: insights from four case studies. Med Teach. 2010;32(12):971–976. 21090950 10.3109/0142159X.2010.497826

[24] Hopkins L, Hampton BS, Abbott JF, et al To the point: medical education, technology, and the millennial learner. Am J Obstet Gynecol. 2018;218(2):188–192. 28599897 10.1016/j.ajog.2017.06.001

[25] Han H, Nelson E, Wetter N Medical students’ online learning technology needs. Clin Teach. 2014;11(1):15–19. 24405913 10.1111/tct.12092

[26] Haferkamp N, Eimler SC, Papadakis A-M, et al Men are from Mars, women are from Venus? Examining gender differences in self-presentation on social networking sites. Cyberpsychology, Behavior, and Social Networking. 2012;15(2):91–98. 10.1089/cyber.2011.0151 22132897

[27] Thompson SH, Lougheed E Frazzled by Facebook? An exploratory study of gender differences in social network communication among undergraduate men and women. Coll Stud J. 2012;46(1): 88–98.

[28] Forgie SE, Duff JP, Ross S Twelve tips for using Twitter as a learning tool in medical education. Med Teach. 2013;35(1):8–14. 23259608 10.3109/0142159X.2012.746448

[29] Davis WM, Ho K, Last J Advancing social media in medical education. Can Med Assoc J. 2015;187(8):549–550. 25852033 10.1503/cmaj.141417 PMC4435861

[30] Cao Y, Ajjan H, Hong P Using social media applications for educational outcomes in college teaching: a structural equation analysis. Brit J Educ Technol. 2013;44(4):581–593.

[31] Adler PA, Adler P The cyber worlds of self‐injurers: deviant communities, relationships, and selves. Symb Interact. 2008;31(1):33–56.

[32] Grinter RE, Palen L, Eldridge M Chatting with teenagers: considering the place of chat technologies in teen life. ACM Trans Comput Hum Interact. 2006;13(4):423–447.

[33] Ellis Y, Daniels B, Jauregui A The effect of multitasking on the grade performance of business students. Res High Educ. 2010;8:1.

[34] Fried CB In-class laptop use and its effects on student learning. Comput Educ. 2008;50(3):906–914.

[35] Amiel T, Sargent SL Individual differences in Internet usage motives. Comput Human Behav. 2004;20(6):711–726.

[36] Moqbel M, Kock N Unveiling the dark side of social networking sites: personal and work-related consequences of social networking site addiction. Info Manag. 2017.55(1): 109–119.

[37] Habitual VA Facebook use and its impact on getting deceived on social media. J Comput Mediat Commun. 2015;20(1):83–98.

[38] Wilson K, Fornasier S, White KM Psychological predictors of young adults’ use of social networking sites. Cyberpsychol Behav Soc Netw. 2010;13(2):173–177. 20528274 10.1089/cyber.2009.0094

[39] Andreassen CS Online social network site addiction: a comprehensive review. Curr Addict Rep. 2015;2(2):175–184.

[40] Guraya SY, Norman RI, Roff S Exploring the climates of undergraduate professionalism in a Saudi and a UK medical school. Med Teach. 2016;38(6):630–632. 27007746 10.3109/0142159X.2016.1150987

[41] Guraya SY, Guraya SS, Almaramhy HH The legacy of teaching medical professionalism for promoting professional practice: a systematic review. Biomed Pharmacol J. 2016;9(2):809–817.

[42] McDonald P, Thompson P Social media (tion) and the reshaping of public/private boundaries in employment relations. Inter J Manag Rev. 2016;18(1):69–84.

